# Primary tumor resection for asymptomatic colorectal cancer patients with synchronous unresectable metastases: a meta-analysis of randomized controlled trials and case-matched studies

**DOI:** 10.1007/s00423-024-03414-9

**Published:** 2024-08-06

**Authors:** Jun Huang, Jiahao Zhou, Ping Zhang, Qingbin Wu, Ziqiang Wang

**Affiliations:** 1 Colorectal Cancer Center, Department of General Surgery, West China Hospital, Sichuan University, No. 37, Guo Xue Xiang, Chengdu, 610041 China; 2 Emergency Medicine Department of West China Hospital, Sichuan University, Chengdu, 610041 China; 3 West China Medical School, Sichuan University, Chengdu, 610041 China

**Keywords:** Metastatic colorectal cancer, Primary tumor resection, Survival, Meta-analysis

## Abstract

**Purpose:**

The value of upfront primary tumor resection (PTR) for asymptomatic unresectable metastatic colorectal cancer (mCRC) patients remains contentious. This meta-analysis aimed to assess the prognostic significance of upfront PTR for asymptomatic unresectable mCRC.

**Methods:**

A systematic literature search was performed on June 21st, 2024. To minimize the bias and ensure robust evidence, only randomized controlled trials (RCTs) and case-matched studies (CMS) that compared PTR followed by chemotherapy to chemotherapy alone were included. The primary outcome was overall survival (OS), while cancer-specific survival (CSS) served as the secondary outcome.

**Results:**

Eight studies (three RCTs and five CMS) involving 1221 patients were included. Compared to chemotherapy alone, upfront PTR followed by chemotherapy did not improve OS (hazard ratios [HR] 0.91, 95% confidence interval [CI] 0.79–1.04, *P* = 0.17), but was associated with slightly better CSS (HR 0.59, 95% CI 0.40–0.88, *P* = 0.009).

**Conclusions:**

The current limited evidence indicates that upfront PTR does not improve OS but may enhance CSS in asymptomatic unresectable mCRC patients. Ongoing trials are expected to provide more reliable evidence on this issue.

**Supplementary Information:**

The online version contains supplementary material available at 10.1007/s00423-024-03414-9.

## Introduction

Colorectal cancer (CRC) is the third most commonly diagnosed cancer and the second leading cause of cancer-related mortality worldwide, with an estimated 1.9 million incidence cases and 0.9 million deaths in 2020 [1]. At the time of diagnosis, approximately 15-30% of CRC patients present with synchronous distant metastases, more than 80% of which are unresectable [2, 3]. While upfront primary tumor resection (PTR) is recommended for unresectable metastasis CRC (mCRC) patients with tumor-related symptoms like bleeding, perforation, or obstruction, its value for asymptomatic patients remains contentious.

Several retrospective studies compared PTR followed by chemotherapy to chemotherapy alone for asymptomatic unresectable mCRC, yielding conflicting results. For instance, Matsuda et al. and Ahmed et al. reported improved patient survival with PTR followed by chemotherapy [4, 5], while two other studies did not [6, 7]. The disparate results may be partly explained by the heterogeneity among mCRC patients and the selection bias inherent in the retrospective design. Recent randomized controlled trials (RCTs) also failed to demonstrate a survival benefit of PTR followed by chemotherapy, albeit prematurely terminated due to insufficient patient enrollment [8–10]. Therefore, there is still no consensus on whether upfront PTR should be performed in patients with asymptomatic unresectable mCRC. To address this, we conducted a meta-analysis, including only RCTs and case-matched studies (CMS), to assess the prognostic value of upfront PTR for these patients.

## Materials and methods

The current meta-analysis was conducted according to the guidance of the preferred items for systematic reviews and meta-analyses (PRISMA) statement [11].

### Study selection

A systematic search with no limits was performed in four electronic databases, including PubMed, Embase, Web of Science, and Cochrane Central Register of Controlled Trails. The search algorithm included “metastases”, “colorectal cancer”, and “asymptomatic” with mapping terms. The detailed search of each database is shown in Table S1. The initial search was conducted in June 2021 and the latest update was performed on June 21st, 2024.

### Inclusion and exclusion criteria

In this meta-analysis, we included RCTs and CMS that compared PTR followed by chemotherapy (PTR group) versus chemotherapy alone (non-PTR group) for patients with asymptomatic unresectable mCRC. Only published data were considered for analysis. The exclusion criteria were as follows: (1) studies involving patients with primary tumor-related symptoms that were defined as conditions requiring emergency intervention (i.e. bleeding, perforation, and obstruction). (2) studies that did not report survival outcomes; (3) studies involving patients receiving induction chemotherapy prior to PTR; (4) single-armed studies; (5) case reports, conference abstracts, study protocols, letters, comments, reviews, or meta-analysis. For studies including overlap patients of the same center, the one with longer follow-up periods was included.

### Data extraction and outcomes

Two investigators independently reviewed all identified studies and extracted items from the included studies. Disagreements were resolved with the assistance of a third reviewer. The following items of all included studies were extracted: the first author’s name, year of publication, study design, follow-up period, interventions, patients of each arm, age, male gender, primary tumor and metastatic site, palliative treatment regimens, and survival outcomes. The primary outcome was overall survival (OS), which was defined as the time from random assignment or initial treatment (PTR or systemic treatment) to death because of any reason or the last censored date during follow-up. The secondary outcome was cancer-specific survival (CSS), which was measured from random assignment or initial treatment to CRC-related death.

### Quality assessment

The methodological quality of the included RCTs was evaluated using the Cochrane Collaboration’s tool, which assesses seven domains: random sequence generation, allocation concealment, blinding of participants and personnel, blinding of outcome assessment, incomplete outcome data, selective reporting, and other bias [12]. For each of the domains, the studies were categorized as high, low, and uncertain risk. The methodological quality of CMS was assessed using the Newcastle-Ottawa scale, which was consisted of eight items and categorized into three dimensions (selection, comparability, and exposure), with a total of nine scores (< 5 scores, low quality; 5–7 scores, middle quality; 8–9 scores, high quality) [13].

### Statistical analysis

To compare survival outcomes between the PTR and non-PTR groups, either the fixed-effects model (for I^2^ of Higgins I^2^-test ≤ 50%) or the random-effects model (for I^2^ > 50%) was employed to calculate pooled hazard ratios (HRs) with a 95% confidence interval (CI) for OS and CSS using the Review Manager (version 5.3) software. HRs with 95% CIs were directly extracted from the included studies. If studies did not report HRs and/or 95% CIs, the Engauge Digitizer software was used to extract data from Kaplan-Meier curves according to the method developed by Parmar and colleagues [14]. Subsequently, these data were used to recalculate HRs and 95% CIs online according to the method proposed by Liu et al. [15] (https://www.trialdesign.org/one-page-shell.html#IPDfromKM). Subgroup analysis was performed according to the study design. The sensitivity analysis was conducted with the leave-one-out approach. Publication bias was assessed by graphical exploration with Begg’s funnel plots. A P value < 0.05 was considered statistically significant.

## Results

### Study selection

**Fig. 1:**
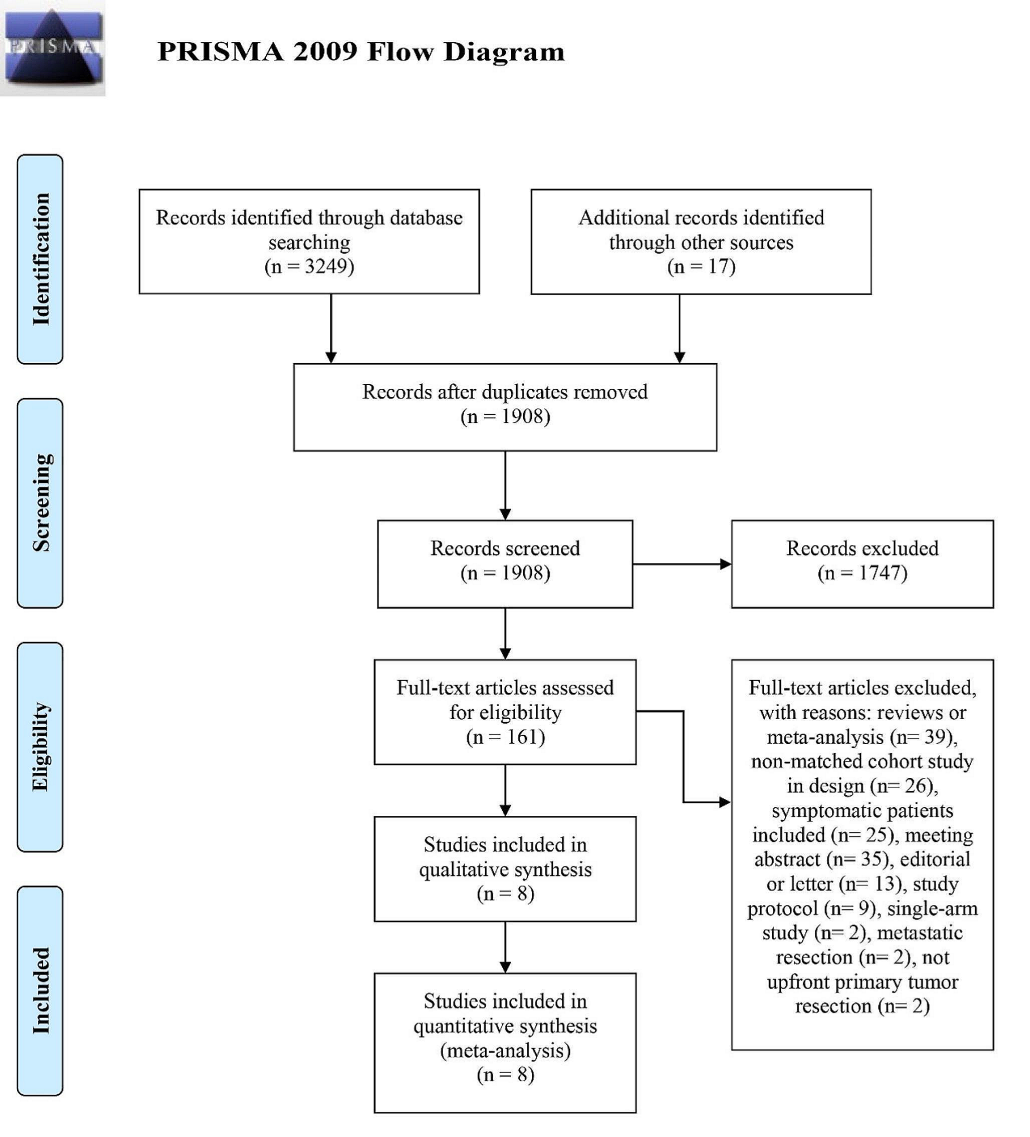
PRISMA-flow diagram

The PRISMA diagram of this meta-analysis is depicted in Fig. 1. After removing duplicates and including additional records identified through references in related articles, a total of 1908 studies were retrieved. Through title and abstract screening, 1747 irrelevant studies were excluded, leaving 161 studies for full-text assessment. Subsequently, 153 studies were further excluded based on predefined criteria, as illustrated in Fig. 1. Ultimately, eight studies met the eligibility criteria and were included in the meta-analysis, comprising 3 RCTs [8–10] and 5 cm [16–20]. A total of 1221 patients were enrolled, with 629 in the PTR group and 592 in the non-PTR group.

### Study characteristics and quality assessment

The baseline characteristics of included studies are summarized in Tables 1 and 2. All studies encompassed patients diagnosed with colon and rectal cancer, except Rahbari et al. [8], who focused solely on colon cancer. Notably, Benoist et al. [20] exclusively recruited patients with liver metastases, while other studies did not impose restrictions regarding metastatic sites. Palliative treatment regimens were mostly based on oxaliplatin or irinotecan, often combined with targeted agents, which was in line with the contemporary standard therapy protocols. All RCTs exhibited moderate risks of bias (Fig. S1), and the other five CMS had middle methodological quality (Table S2).

### OS

**Fig. 2:**
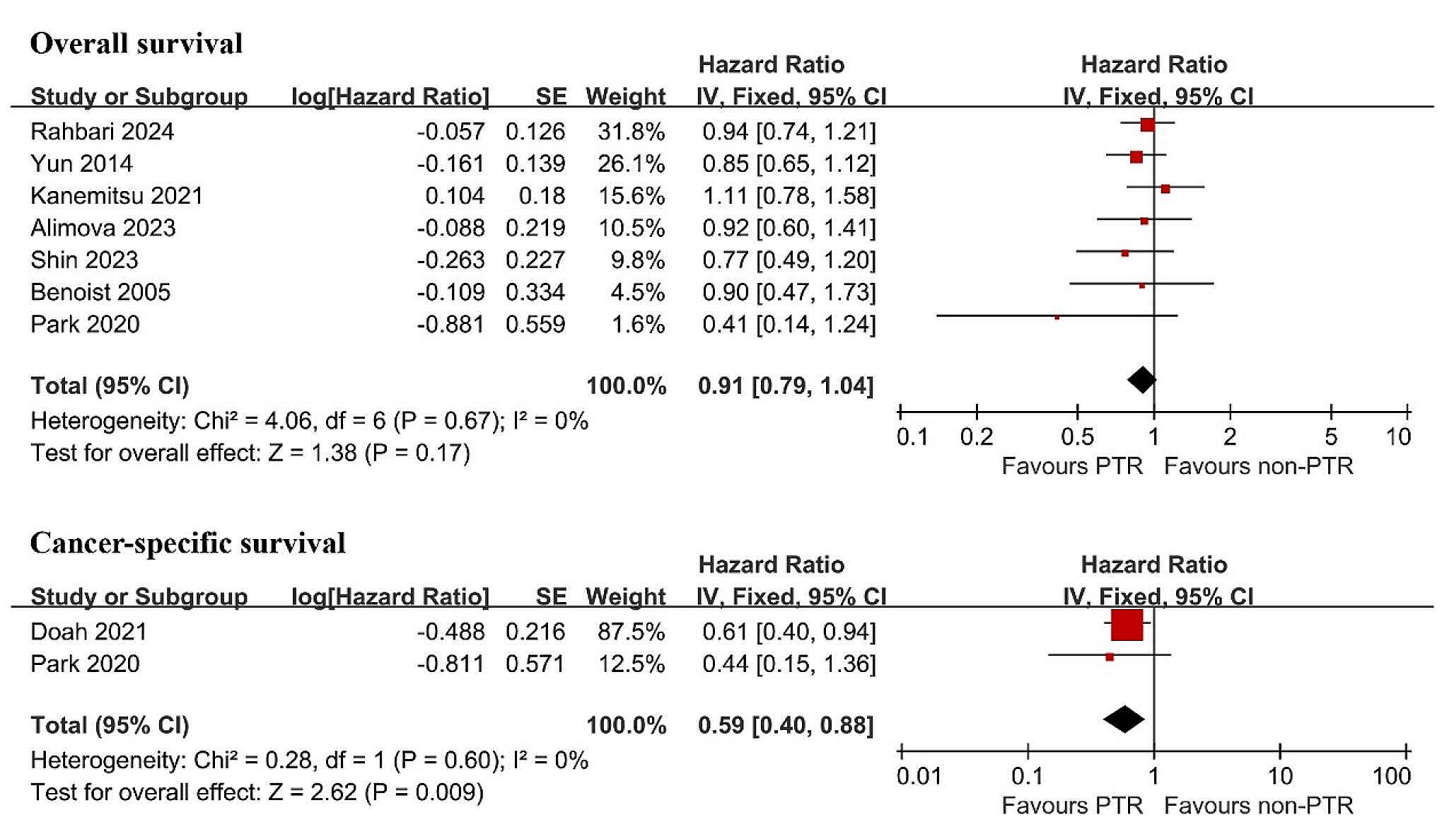
Comparisons of overall and cancer-specific survival. PTR primary tumor resection

Seven out of eight articles reported OS and were included in the meta-analysis [8–10, 16, 17, 19, 20]. Overall, the 1-year and 2-year OS rates were 59-84% and 29-70% in the PTR group and 59-86% and 23-56% in the non-PTR group, respectively (Table 2). The fixed-effects model analysis indicated that OS was similar between the PTR and non-PTR groups (HR 0.91, 95% CI 0.79–1.04, *P* = 0.17) (Fig. 2). Sensitivity analysis was conducted and the results remained consistent, which showed great interstudy homogeneity (I^2^ = 0%) (Table 3).

### CSS

Two out of eight articles reported CSS and were included in the meta-analysis [10, 18]. Overall, the 1-year and 2-year CSS rates were 72-87% and 46-72% in PTR group and 62-63% and 29-47% in the non-PTR group, respectively (Table 2). The fixed-effects model analysis revealed that, compared to the non-PTR group, the PTR group was associated with slightly better CSS (HR 0.59, 95% CI 0.40–0.88, *P* = 0.009) (Fig. 2).

### Subgroup analysis

**Fig. 3:**
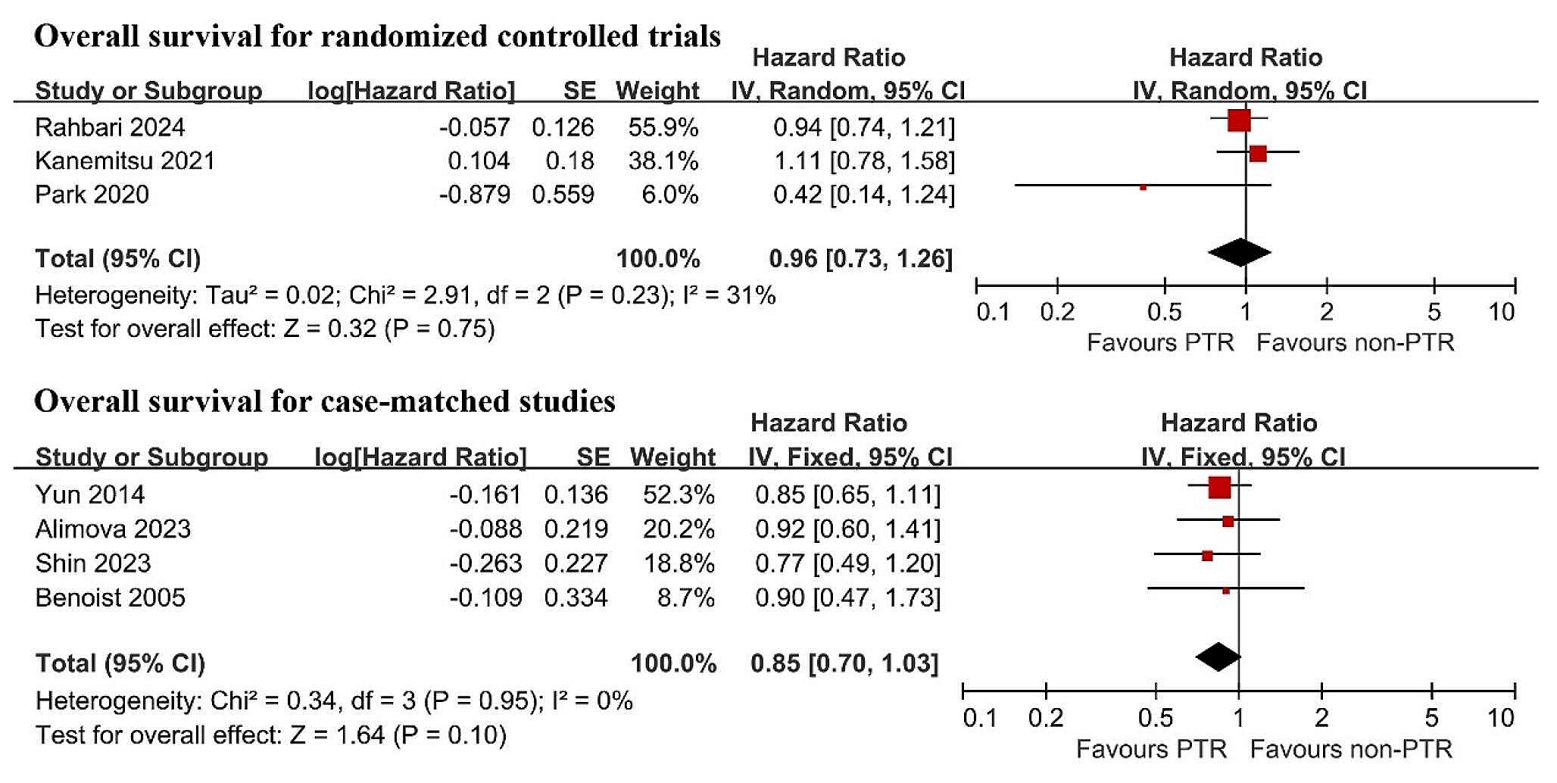
Subgroup analysis of overall survival according to the study design. PTR primary tumor resection

The subgroup analysis for OS was performed according to the study design (RCTs and CMS). Pooled analysis of RCTs showed no significant difference in OS between the PTR and non-PTR groups (HR 0.96, 95% CI 0.73–1.26, *P* = 0.75), and this finding was consistent in the CMS (HR 0.85, 95% CI 0.70–1.03, *P* = 0.10) (Fig. 3).

### Publication bias

**Fig. 4:**
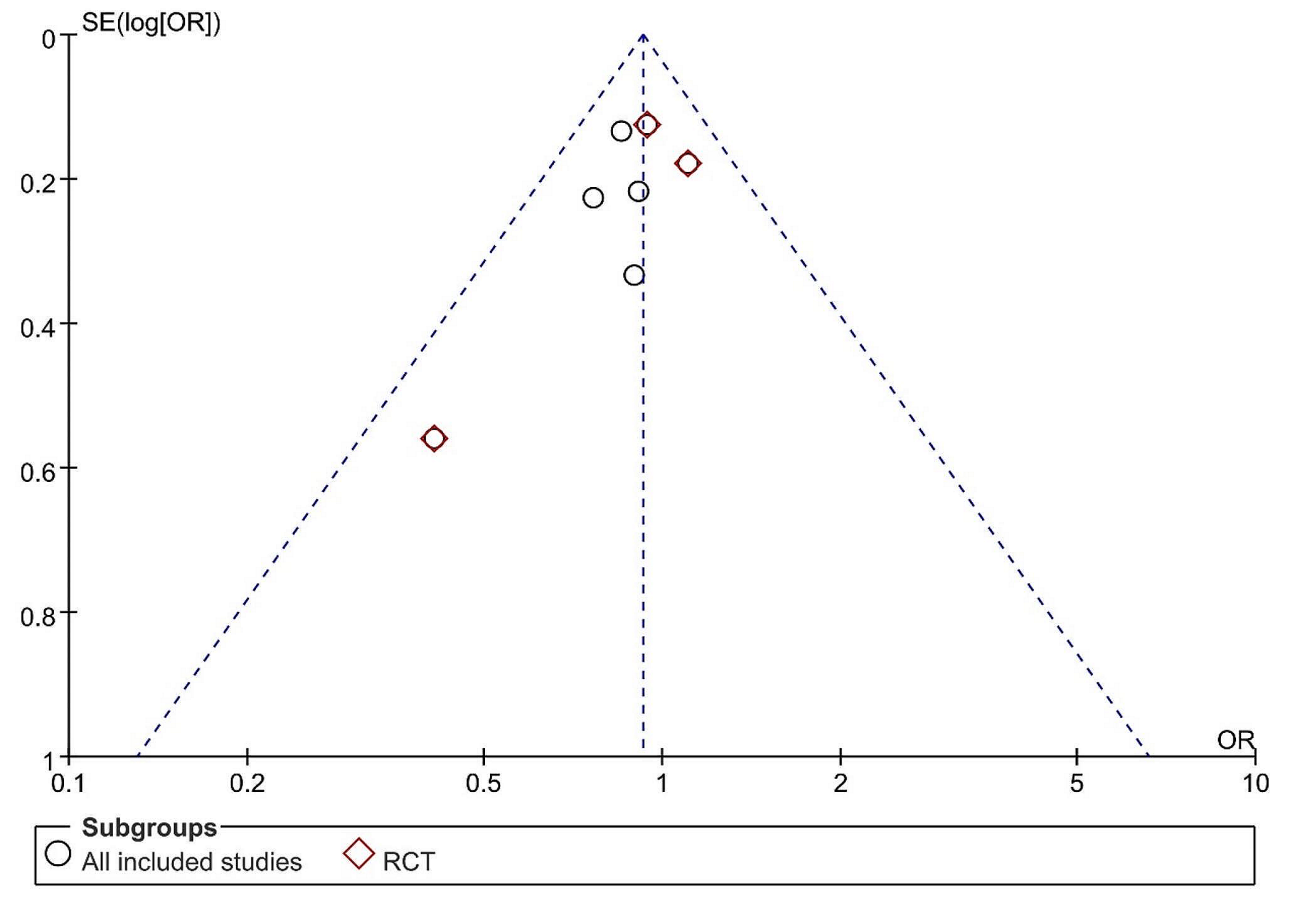
Publication bias

Publication bias was assessed based on OS (Fig. 4). The funnel plots were found to be symmetric, indicating that publication bias did not exist.

**Table 1:** Characteristics of included studies

Study	Study design	Median fellow-up (months)	Group	Patients	Age ^*^ (years)	Male gender (%)	Primary site (rectum, %)	Metastatic sites	Palliative treatment regimens	Survival outcomes	Quality ^#^
Rahbari et al. 2024 [8]	RCT	36.7	PTR	187	69 (61–75)	67.4	0	Liver/lung/distant lymph node/peritoneum/bone	FOLFIRI/FOLFOX ± BEV/CET	OS	Moderate
			Non-PTR	206	69 (62–75)	66.0	0	Liver/lung/distant lymph node/peritoneum/bone	FOLFIRI/FOLFOX ± BEV/CET		
Park et al. 2020 [10]	RCT	15	PTR	26	62 ± 12	80.8	26.9	Liver/lung/peritoneum	FOLFIRI ± BEV/CET	OS, CSS	Moderate
			Non-PTR	22	59 ± 12	54.5	13.6	Liver/lung	FOLFIRI ± BEV/CET		
Kanemitsu et al. 2021 [9]	RCT	22.1	PTR	81	65 (59–69)	56.0	7.4	Liver/lung/distant lymph node/peritoneum	FOLFOX/XELOX + BEV	OS, PFS	Moderate
			Non-PTR	84	65 (59–71)	54.0	7.1	Liver/lung/distant lymph node/peritoneum	FOLFOX/XELOX + BEV		
Shin et al. 2023 [16]	Retrospective	18	PTR	42	60 (34–84)	73.8	NA	NA	FOLFIRI/FOLFOX ± BEV/CET	OS	Middle
			Non-PTR	42	NA	NA	NA	NA	FOLFIRI/FOLFOX ± BEV/CET		
Alimova et al. 2023 [17]	Retrospective	23.2	PTR	50	60.5 (29–83)	50.0	44.0	Liver/lung/retroperitoneal lymph node	FOLFIRI/FOLFOX ± BEV/CET	OS	Middle
			Non-PTR	50	58 (33–80)	52.0	48.0	Liver/lung/retroperitoneal lymph node	FOLFIRI/FOLFOX ± BEV/CET		
Doah et al. 2021 [18]	Retrospective	18	PTR	98	69 (58–77)	50.0	43.9	Liver/lung/distant lymph node/peritoneum/bone/ovary	FOLFOX/FOLFIRI ± BEV/CET	CSS	Middle
			Non-PTR	48	67 (62–75)	60.4	43.8	Liver/lung/distant lymph node/peritoneum/bone/ovary	FOLFOX/FOLFIRI ± BEV/CET		
Yun et al. 2014 [19]	Retrospective	16	PTR	113	59 (23–87)	39.4	38.1	Liver/lung/distant lymph node/peritoneum/bone	FOLFIRI/FOLFOX ± BEV/CET	OS	Middle
			Non-PTR	113	60 (25–77)	39.8	30.0	Liver/lung/distant lymph node/peritoneum/bone	FOLFIRI/FOLFOX ± BEV/CET		
Benoist et al. 2005 [20]	Retrospective	NA	PTR	32	63 ± 13	59.4	28.1	Liver	FOLFIRI/FOLFOX	OS	Middle
			Non-PTR	27	61 ± 12	66.7	14.8	Liver	FOLFIRI/FOLFOX		

^*^ Ages are shown as mean ± standard deviation or median with interquartile range, ^#^ quality of RCTs and retrospective studies were assessed according to the Cochrane Collaboration’s tool and Newcastle-Ottawa scale, respectively. *RCT* randomized controlled trial, *PTR* primary tumor resection, *NA* not available, *BEV* Bevacizumab, *CET* Cetuximab, *OS* overall survival, *CSS* cancer-specific survival, *PFS* progression-free survival

**Table 2:** Survival outcomes of included studies

Study	Group	OS rate (%)			CSS rate (%)			PFS rate (%)		
		1-year	2-year	3-year	1-year	2-year	3-year	1-year	2-year	3-year
Rahbari et al. 2024 [8]	PTR	59.2	30.8	17.5	—	—	—	—	—	—
	Non-PTR	65.9	39.0	20.4	—	—	—	—	—	—
Park et al. 2020 [10]	PTR	83.9	69.5	—^*^	87.3	72.3	—^*^	—	—	—
	Non-PTR	58.7	44.8	—^*^	62.6	47.1	—^*^	—	—	—
Kanemitsu et al. 2021 [9]	PTR	76.5	54.1	35.1	—	—	—	45.7	12.5	5.5
	Non-PTR	85.7	55.8	34.9	—	—	—	53.6	13.7	6.9
Shin et al. 2023 [16]	PTR	78.0	43.8	28.9	—	—	—	—	—	—
	Non-PTR	71.2	34.8	20.7	—	—		—	—	—
Alimova et al. 2023 [17]	PTR	79.8	59.0	42.1	—	—	—	—	—	—
	Non-PTR	81.5	55.8	34.0	—	—	—	—	—	—
Doah et al. 2021 [18]	PTR	—	—	—	71.8	45.5	17.8	—	—	—
	Non-PTR	—	—	—	61.8	28.6	5.5	—	—	—
Yun et al. 2014 [19]	PTR	67.0	29.0	7.5	—	—	—	—	—	—
	Non-PTR	59.7	23.2	8.2	—	—	—	—	—	—
Benoist 2005 [20]	PTR	77.8	43.8	—^*^	—	—	—	—	—	—
	Non-PTR	69.8	41.6	—^*^	—	—	—	—	—	—

*PTR* primary tumor resection, *OS* overall survival, *CSS* cancer-specific survival, *PFS* progression-free survival, — not available

^*^ The follow-up ended beforehand

**Table 3:** Sensitivity analysis of overall survival

Sensitivity analysis	I^2^	Model	HR	95% CI	*P* value
Rahbari et al. 2024 removed	0%	Fixed	0.89	0.75–1.05	0.17
Shin et al. 2023 removed	0%	Fixed	0.92	0.80–1.07	0.28
Alimova et al. 2023 removed	0%	Fixed	0.91	0.78–1.05	0.19
Park et al. 2020 removed	0%	Fixed	0.92	0.80–1.06	0.23
Kanemitsu et al. 2021 removed	0%	Fixed	0.87	0.75–1.02	0.08
Yun et al. 2014 removed	0%	Fixed	0.93	0.79–1.09	0.36
Benoist et al. 2005 removed	0%	Fixed	0.91	0.79–1.05	0.18
All studies	0%	Fixed	0.91	0.79–1.04	0.17

*HR* hazard ratios, *CI* confidence interval

## Discussion

This meta-analysis aimed to assess the prognostic significance of PTR followed by chemotherapy for patients with asymptomatic unresectable mCRC. To minimize the bias and ensure robust evidence, only RCTs and that compared PTR followed by chemotherapy to chemotherapy alone were included. Evidence from three RCTs and five CMS were combined, indicating that PTR followed by chemotherapy did not improve OS compared to chemotherapy alone, but may enhance CSS in unresectable mCRC patients.

Upfront PTR is recommended for unresectable mCRC patients with primary tumor-related symptoms; nevertheless, the value of PTR in asymptomatic patients remains contentious. Undoubtedly, PTR in asymptomatic patients can prevent primary tumor-related complications that might arise during chemotherapy [21]. Additionally, a reduction in tumor burden by PTR could increase treatment response and theoretically confer survival benefits [22, 23]. Conversely, PTR delays the initiation of systemic treatment, especially for patients experiencing postoperative complications, potentially accelerating tumor progression due to its impact on physical condition [24].

Several large cohort studies have compared PTR followed by chemotherapy to chemotherapy alone. Population-based data from various public databases, employing propensity score matching analysis, indicated that PTR was associated with better OS [25–27]; however, patients with symptoms were not strictly excluded in these studies as some clinical information was not available in the database. In addition, van Rooijen et al. enrolled patients from 8 RCTs and demonstrated improved survival in synchronous mCRC patients after PTR, but notable differences existed in baseline characteristics between the groups and they did not use case-matched method [28]. Fanotto et al. conducted a pooled analysis based on individual patient data from two RCTs, and extended PFS and OS were observed in patients who underwent upfront PTR, despite a lower metastatic burden in this group [29]. In contrast to these studies, three recent RCTs failed to demonstrate a survival benefit of PTR followed by chemotherapy [8–10]. Rahbari et al. pooled the data of two RCTs, the SYNCHRONOUS and the CR4 trials, comprising 393 patients in the combined cohort, with a median follow-up of 36.7 months—the largest sample size and longest follow-up period in this context [8]. They indicated PTR did not confer an overall advantage (median OS was 16.7 versus 18.6 months, HR 0.94, 95% CI 0.74–1.21, *P* = 0.65), and the absence of OS difference was consistently confirmed by per-protocol analyses within their respective cohort [8]. Similarly, the JCOG1007 trial enrolled 165 patients and demonstrated comparable OS median OS was 25.9 versus 26.7 months, HR 1.10, 95% CI 0.76–1.59) [9]. The other Korean trial only included 48 patients, showing similar two-year OS rates (69.5% vs. 44.8%, *P* = 0.058), but a significantly higher two-year CSS rate in the PTR group (72.3% vs. 47.1%, *P* = 0.049) [10]. The results of our study were consistent with these RCTs.

Several similar trials have not yet published their final results, including the CAIRO4 trial (ClinicalTrials.gov Identifier: NCT01606098), the ORCHESTRA trial (ClinicalTrials.gov identifier: NCT01792934) and a Chinese trial (ClinicalTrials.gov identifier: NCT04416854).At the 2023 Annual Meeting of the American Society of Clinical Oncology (ASCO), Koopman, one of the principal investigators of the CAIRO4 trial, launched the latest results from 204 patients [30]. With a median follow-up period of 63.6 months, it ultimately indicated that upfront PTR did not yield a significant survival benefit compared to systemic treatment alone (median OS was 20.5 versus 18.3 months, *P* = 0.345; median PFS was 10.1 versus 10.1 months, *P* = 0.805) [30]. Recently, at the 2024 Annual Meeting of ASCO, Gootjes presented that additional tumor debulking alongside standard first-line palliative systemic therapy failed to improve OS for patients with multiorgan mCRC [31]. These unpublished data of large ongoing RCTs also support our findings and we expect their comprehensive publication would provide more reliable evidence on this issue.

It is noteworthy that all published RCTs were brought to an early termination. According to Rahbari’s study [8], data of two individual trials (the SYNCHRONOUS and the CCRe-IV trials) were pooled due to slow recruitment, with the aim to accelerate the reporting of primary end point. The final cohort included 295 patients from the SYNCHRONOUS trial and 98 patients from the CCRe-IV trial, which were much less than prespecified sample sizes [32, 33]. The JCOG1007 trial was terminated prematurely at the interim analysis because of futile results between the two groups [9]. Likewise, the Korean trial ended early due to insufficient recruitment and abrupt cessation of research funding, therefore an extremely small sample size [10]. Furthermore, they implied actual difficulties in convincing patients to accept random assignment of surgery interventions versus non-surgery interventions [9, 10].

Despite overall futility, CSS was found to be better in the PTR group. After applying the inverse probability of treatment weighting (IPTW) method to minimize selection bias, Doah et al. [18] conducted a survival analysis of 146 patients and demonstrated that PTR was associated with prolonged CSS (HR 0.61, 95% CI 0.40–0.94, *P* = 0.024), which comprise about 87.5% of statistical power in this meta-analysis, while the other Korean trail account for only 12.5% [10]. Given the limited number of studies included, we discreetly concluded that PTR may improve CSS. This may be attributed to the reduction of tumor burden and prevention of potential tumor-related complications during chemotherapy achieved by PTR. Meanwhile, due to the inherent limitation of retrospective studies, selection bias is inevitable; in other words, patients selected for PTR might have had better overall health or fewer comorbidities even when analysis were adjusted for cofounders. Furthermore, PTR could provide better local control of the disease, which is crucial in preventing cancer-related deaths caused by local progression of the primary tumor, despite the overall systemic burden of the disease remaining unchanged.

Recently, two meta-analyses have assessed the value of PTR for asymptomatic unresectable mCRC, but the result was debated. One included eight studies and the pooled result showed that the PTR group had better OS compared to the non-PTR group [34], whereas the other did not [35]. Careful interpretation of the results of these meta-analyses is warranted due to their inclusion of low-quality retrospective studies. Our meta-analysis, however, only included RCTs and CMS to minimize the bias, thus providing more reliable evidence.

The reason why PTR may not improve OS in asymptomatic patients is unclear. We hypothesized that, in contrast to symptomatic patients, asymptomatic patients may have a lower tumor burden, and the primary tumor may not be the major source of tumor progression. In addition, there is a likelihood that the specific subset of patients who respond favorably to systemic therapy may benefit from PTR. Huang et al. denoted in a recent RCT that PTR failed to confer survival benefits for colon cancer patients with unresectable metastases and yet shortened the progression-free survival (PFS) in patients who did not achieve a partial response (PR) to induction chemotherapy [24]. Perhaps more precise patient selection should be considered, taking into account factors such as tumor response and primary tumor site. Nevertheless, this hypothesis requires further confirmation. Another possible reason could be that, although PTR may prevent primary tumor-related complications, the severe intestinal complications that necessitate surgery during chemotherapy were low (less than 15%) [9, 36]. Additionally, surgery-related complications may delay the initiation of chemotherapy. Moreover, the CAIRO4 trial even reported an increased 60-day mortality with upfront PTR (11% vs. 3%, *P* = 0.03) [37]. Taken together, it is not surprising that PTR does not improve OS in asymptomatic patients.

To the best of our knowledge, this meta-analysis provided the most robust evidence to evaluate the value of PTR for asymptomatic unresectable mCRC. Our results denoted that PTR followed by chemotherapy did not improve OS compared to chemotherapy alone, but was associated with slightly better CSS. Nevertheless, several limitations need to be acknowledged. Firstly, the present meta-analysis included a limited number of articles (three RCTs and five CMS). We will update the results as more evidence is accumulated with time. Secondly, certain factors that might influence survival outcomes were not reported or varied across studies. For example, the biological behavior of mCRC patients with left colon cancer (LCC) and rectal cancer was better than that of patients with right colon cancer (RCC). Zhang et al. found that for LCC patients, PTR prolonged the median OS time; however, for right-side colon cancer patients, PTR conferred no benefit [38]. Similarly, a population-based study on 35,690 patients from two National Cancer Registries showed that upfront PTR improved median OS in RCC, LCC, and rectal cancer, but resulted in higher 30-day mortality after surgery in RCC [39]. Regarding rectal cancer, clinical complexity increases as PTR poses challenges with increased morbidity, therefore conservative treatment alternatives like diversion or local radiotherapy have typically been preferred. In the present study, because no included studies stratified patients according to primary tumor site, we were unable to perform this subgroup analysis. This was the case with other important variables, such as site and number of metastases, RAS genes status, and chemotherapy protocols. Thirdly, as shown in the RECUT trial, induction chemotherapy before PTR enhanced the PFS compared to upfront PTR, whereas no significant difference in OS was noted [40]. Another CMS demonstrated the conclusion in line [41]. However, the surgery timing was not addressed in this study. Last but not least, although funnel plots were found to be symmetric, potential publication bias might still exist because some RCTs registered on ClinicalTrials.gov were prematurely terminated due to insufficient patient enrollment and were not published. Despite these limitations, our meta-analysis provides evidence that upfront PTR cannot be endorsed as the standard of care at present.

The current limited evidence indicates that upfront PTR does not improve OS but may enhance CSS in asymptomatic unresectable mCRC patients. Ongoing trials are expected to provide more reliable evidence on this issue.

### Electronic supplementary material

Below is the link to the electronic supplementary material.

Supplementary Material 1

Supplementary Material 2

Supplementary Material 3

Supplementary Material 4

## Data Availability

Data is provided within the manuscript.
