# Supplementary figures and images for: Primary tumor resection for asymptomatic colorectal cancer patients with synchronous unresectable metastases: a meta-analysis of randomized controlled trials and case-matched studies
Source: Langenbecks Arch Surg. 2024 Aug 6;409(1):242. doi: 10.1007/s00423-024-03414-9 (PMC11303460; doi:10.1007/s00423-024-03414-9)

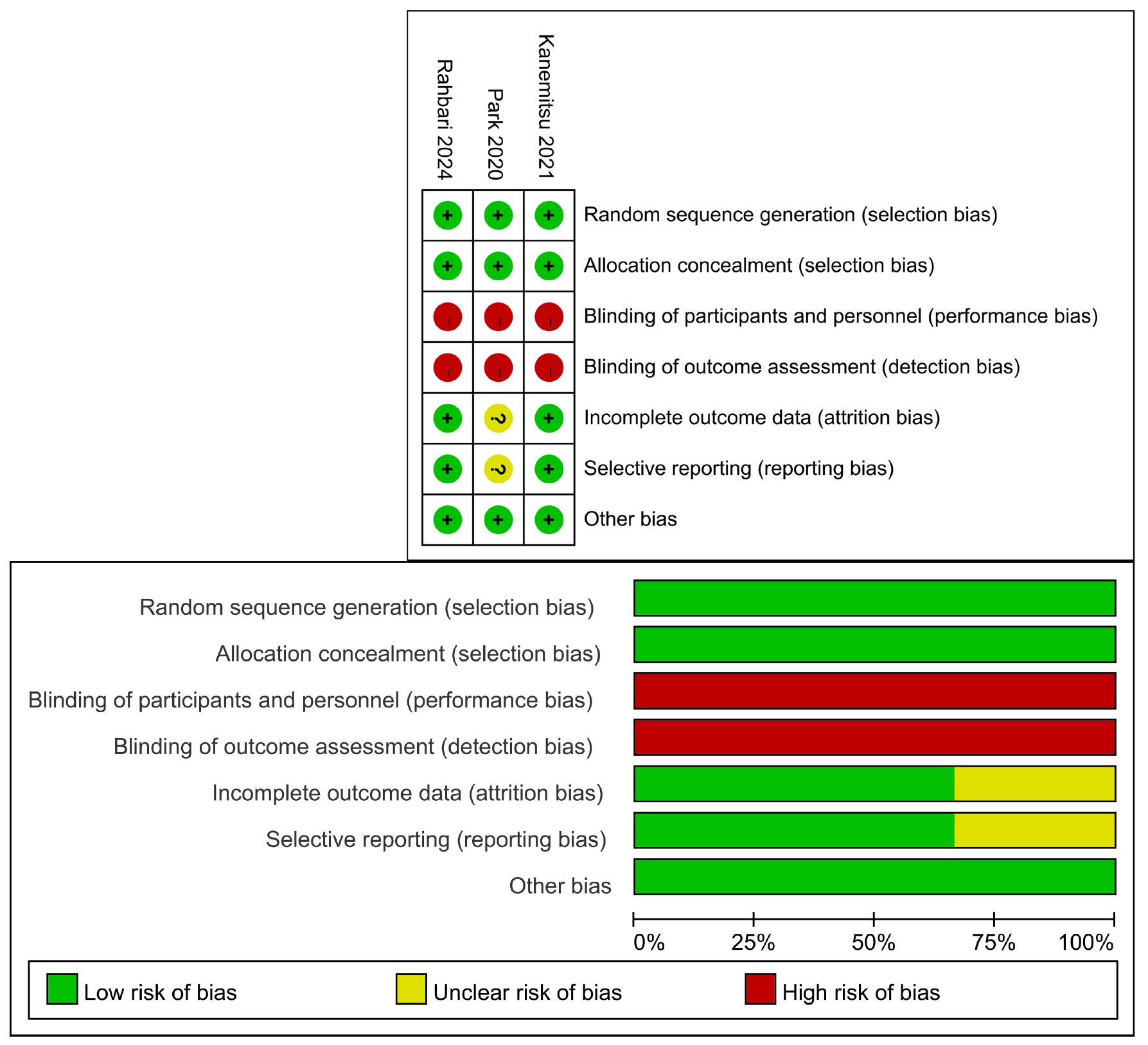

Supplement: Supplementary file 1 — Supplementary Material 1 [file 423_2024_3414_MOESM1_ESM.tif]
