# Supplementary material for: Primary tumor resection for asymptomatic colorectal cancer patients with synchronous unresectable metastases: a meta-analysis of randomized controlled trials and case-matched studies
Source: Langenbecks Arch Surg. 2024 Aug 6;409(1):242. doi: 10.1007/s00423-024-03414-9 (PMC11303460; doi:10.1007/s00423-024-03414-9)
Supplement: Supplementary file 2 — Supplementary Material 2 [file 423_2024_3414_MOESM2_ESM.docx]

**Table S1** Search strategy of the systematic review.

| Database and search strategy | | Items |
| --- | --- | --- |
| **PubMed (up to June 21^st^, 2024)** | | |
| 1 | Metastases OR "stage IV" OR metastasis OR metastatic | 1753170 |
| 2 | Colon cancer OR colon cancers OR colon carcinoma OR colon carcinomas OR rectal cancer OR rectal cancers OR rectal carcinoma OR rectal carcinomas OR colorectal cancer OR colorectal cancers OR colorectal carcinoma OR colorectal carcinomas | 376286 |
| 3  4 | Symptomless OR asymptomatic OR symptom-free OR "minimally symptomatic" OR "no symptoms" OR "no symptom"  1 AND 2 AND 3 | 223516  995 |
| **Web of Science All Collection (1900 to June 18^th^, 2024)** | | |
| 1 | TS = (Metastases OR "stage IV" OR metastasis OR metastatic) | 1128898 |
| 2 | TS = (Colon cancer OR colon cancers OR colon carcinoma OR colon carcinomas OR rectal cancer OR rectal cancers OR rectal carcinoma OR rectal carcinomas OR colorectal cancer OR colorectal cancers OR colorectal carcinoma OR colorectal carcinomas) | 665724 |
| 3 | TS = (Symptomless OR asymptomatic OR symptom-free OR "minimally symptomatic" OR "no symptoms" OR "no symptom") | 293775 |
| 4 | 1 AND 2 AND 3 | 984 |
| **Embase (via Ovid, 1974 to June 21^st^, 2024)** | | |
| 1 | (Metastases OR "stage IV" OR metastasis OR metastatic). af. | 1173628 |
| 2 | (Colon cancer OR colon cancers OR colon carcinoma OR colon carcinomas OR rectal cancer OR rectal cancers OR rectal carcinoma OR rectal carcinomas OR colorectal cancer OR colorectal cancers OR colorectal carcinoma OR colorectal carcinomas). af. | 454199 |
| 3 | (Symptomless OR asymptomatic OR symptom-free OR "minimally symptomatic" OR "no symptoms" OR "no symptom"). af. | 332291 |
| 4 | 1 AND 2 AND 3 | 1206 |
| **The Cochrane Central Register of Controlled Trails (via Ovid, up to May 2024)** | | |
| 1 | (Metastases OR "stage IV" OR metastasis OR metastatic). af. | 57216 |
| 2 | (Colon cancer OR colon cancers OR colon carcinoma OR colon carcinomas OR rectal cancer OR rectal cancers OR rectal carcinoma OR rectal carcinomas OR colorectal cancer OR colorectal cancers OR colorectal carcinoma OR colorectal carcinomas). af. | 23321 |
| 3 | (Symptomless OR asymptomatic OR symptom-free OR "minimally symptomatic" OR "no symptoms" OR "no symptom"). af. | 16064 |
| 4 | 1 AND 2 AND 3 | 64 |
