# Supplementary material for: Primary tumor resection for asymptomatic colorectal cancer patients with synchronous unresectable metastases: a meta-analysis of randomized controlled trials and case-matched studies
Source: Langenbecks Arch Surg. 2024 Aug 6;409(1):242. doi: 10.1007/s00423-024-03414-9 (PMC11303460; doi:10.1007/s00423-024-03414-9)
Supplement: Supplementary file 3 — Supplementary Material 3 [file 423_2024_3414_MOESM3_ESM.docx]

**Table S2** Methodological quality of retrospective studies according to the Newcastle-Ottawa scale.

| Study | Year | Selection | | | | Comparability | Exposure/outcome | | | Total scores | Quality |
| --- | --- | --- | --- | --- | --- | --- | --- | --- | --- | --- | --- |
|  |  | Represent ativeness of cohort★ | Selection of control cohort★ | Ascertainment of exposure★ | Outcome not present at start★ | Comparability of cohorts★★ | Assessment of outcome★ | Length of follow★ | Adequacy of follow up★ |  |  |
| Shin | 2023 | ★ | ★ | ★ | ★ | ★★ |  | ★ |  | 7 | middle |
| Alimova | 2023 | ★ | ★ | ★ | ★ | ★★ |  | ★ |  | 7 | middle |
| Doah | 2021 | ★ | ★ | ★ | ★ | ★★ |  | ★ |  | 7 | middle |
| Yun | 2014 | ★ | ★ | ★ | ★ | ★★ |  | ★ |  | 7 | middle |
| Benoist | 2005 | ★ | ★ | ★ | ★ | ★ |  | ★ |  | 6 | middle |

<5 scores, low quality; 5-7 scores, middle quality; 8-9 scores, high quality.
