# Supplementary material for: Primary tumor resection for asymptomatic colorectal cancer patients with synchronous unresectable metastases: a meta-analysis of randomized controlled trials and case-matched studies
Source: Langenbecks Arch Surg. 2024 Aug 6;409(1):242. doi: 10.1007/s00423-024-03414-9 (PMC11303460; doi:10.1007/s00423-024-03414-9)
Supplement: Supplementary file 4 — Supplementary Material 4 [file 423_2024_3414_MOESM4_ESM.docx]

**Primary tumor resection for asymptomatic colorectal cancer patients with synchronous unresectable metastases: a meta-analysis of randomized controlled trials and case-matched studies**

Jun Huang ^1,2,4, #^, Jiahao Zhou^1,2,4, #^, Ping Zhang^3,4^, Qingbin Wu^1,2^, Ziqiang Wang ^1,2, *^

^1 Department of General Surgery, West China Hospital, Sichuan University, Chengdu 610041, China.^

^2 Colorectal Cancer Center, West China Hospital, Sichuan University, Chengdu 610041, China.^

^3 Emergency Medicine Department of West China Hospital, Sichuan University, Chengdu 610041, China.^

^4 West China School of Clinical Medicine, Sichuan University, Chengdu 610041, China.^

^# The first two authors contributed equally to this work.^

^* Correspondence to:^

^Dr. Ziqiang Wang, Colorectal Cancer Center, Department of General Surgery, West China Hospital, Sichuan University, No. 37, Guo Xue Xiang, Chengdu, China. Phone: +8602885422480. E-mail address: wangziqiang@scu.edu.cn. ORCID: 0000-0002-2874-1535^

**Corresponding author.** Dr. Ziqiang Wang, Colorectal Cancer Center, Department of General Surgery, West China Hospital, Sichuan University, No. 37, Guo Xue Xiang, Chengdu, China. Phone: +8602885422480. E-mail address: wangziqiang@scu.edu.cn. **ORCID: 0000-0002-2874-1535**

**Supplementary Materials - Index**

| **Supplementary Figures and Tables** |  |
| --- | --- |
| Table S1 Search strategy of the systematic review | *pag. 2* |
| Table S2 Methodological quality of retrospective studies according to the Newcastle-Ottawa scale  Fig S1. Methodological quality of randomized controlled trials according to the Cochrane Collaboration's tool | *pag. 3*  *pag. 4* |
|  |  |

**Supplementary Figures and Tables**

Table S1 Search strategy of the systematic review

| Database and search strategy | | Items |
| --- | --- | --- |
| **PubMed (up to June 21^st^, 2024)** | | |
| 1 | Metastases OR "stage IV" OR metastasis OR metastatic | 1753170 |
| 2 | Colon cancer OR colon cancers OR colon carcinoma OR colon carcinomas OR rectal cancer OR rectal cancers OR rectal carcinoma OR rectal carcinomas OR colorectal cancer OR colorectal cancers OR colorectal carcinoma OR colorectal carcinomas | 376286 |
| 3  4 | Symptomless OR asymptomatic OR symptom-free OR "minimally symptomatic" OR "no symptoms" OR "no symptom"  1 AND 2 AND 3 | 223516  995 |
| **Web of Science All Collection (1900 to June 18^th^, 2024)** | | |
| 1 | TS = (Metastases OR "stage IV" OR metastasis OR metastatic) | 1128898 |
| 2 | TS = (Colon cancer OR colon cancers OR colon carcinoma OR colon carcinomas OR rectal cancer OR rectal cancers OR rectal carcinoma OR rectal carcinomas OR colorectal cancer OR colorectal cancers OR colorectal carcinoma OR colorectal carcinomas) | 665724 |
| 3 | TS = (Symptomless OR asymptomatic OR symptom-free OR "minimally symptomatic" OR "no symptoms" OR "no symptom") | 332291 |
| 4 | 1 AND 2 AND 3 | 984 |
| **Embase (via Ovid, 1974 to June 21^st^, 2024)** | | |
| 1 | (Metastases OR "stage IV" OR metastasis OR metastatic). af. | 1173628 |
| 2 | (Colon cancer OR colon cancers OR colon carcinoma OR colon carcinomas OR rectal cancer OR rectal cancers OR rectal carcinoma OR rectal carcinomas OR colorectal cancer OR colorectal cancers OR colorectal carcinoma OR colorectal carcinomas). af. | 454199 |
| 3 | (Symptomless OR asymptomatic OR symptom-free OR "minimally symptomatic" OR "no symptoms" OR "no symptom"). af. | 326622 |
| 4 | 1 AND 2 AND 3 | 1206 |
| **The Cochrane Central Register of Controlled Trails (via Ovid, up to February 2024)** | | |
| 1 | (Metastases OR "stage IV" OR metastasis OR metastatic). af. | 57216 |
| 2 | (Colon cancer OR colon cancers OR colon carcinoma OR colon carcinomas OR rectal cancer OR rectal cancers OR rectal carcinoma OR rectal carcinomas OR colorectal cancer OR colorectal cancers OR colorectal carcinoma OR colorectal carcinomas). af. | 23321 |
| 3 | (Symptomless OR asymptomatic OR symptom-free OR "minimally symptomatic" OR "no symptoms" OR "no symptom"). af. | 16064 |
| 4 | 1 AND 2 AND 3 | 64 |

**Table S2 Methodological quality of retrospective studies according to the Newcastle-Ottawa scale**

| Study | Year | Selection | | | | Comparability | Exposure/outcome | | | Total scores | Quality |
| --- | --- | --- | --- | --- | --- | --- | --- | --- | --- | --- | --- |
|  |  | Represent ativeness of cohort★ | Selection of control cohort★ | Ascertainment of exposure★ | Outcome not present at start★ | Comparability of cohorts★★ | Assessment of outcome★ | Length of follow★ | Adequacy of follow up★ |  |  |
| Shin | 2023 | ★ | ★ | ★ | ★ | ★★ |  | ★ |  | 7 | middle |
| Alimova | 2023 | ★ | ★ | ★ | ★ | ★★ |  | ★ |  | 7 | middle |
| Doah | 2021 | ★ | ★ | ★ | ★ | ★★ |  | ★ |  | 7 | middle |
| Yun | 2014 | ★ | ★ | ★ | ★ | ★★ |  | ★ |  | 7 | middle |
| Benoist | 2005 | ★ | ★ | ★ | ★ | ★ |  | ★ |  | 6 | middle |

<5 scores, low quality; 5-7 scores, middle quality; 8-9 scores, high quality.

**
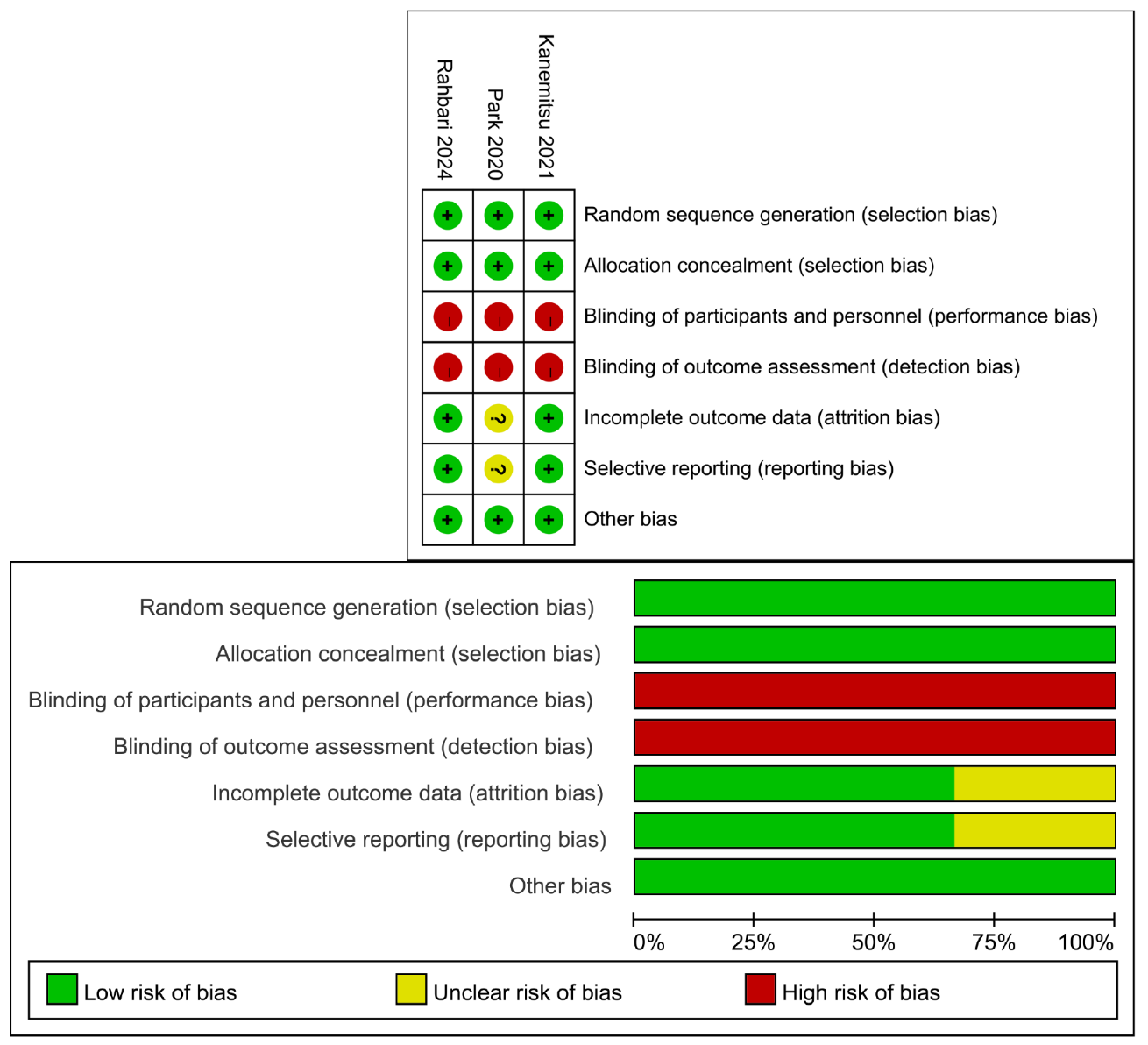
**

**Fig S1. Methodological quality of randomized controlled trials according to the Cochrane Collaboration's tool**
